# Supplementary material for: A bitopic agonist bound to the dopamine 3 receptor reveals a selectivity site
Source: Nat Commun. 2024 Sep 5;15:7759. doi: 10.1038/s41467-024-51993-4 (PMC11377762; doi:10.1038/s41467-024-51993-4)
Supplement: Supplementary file 1 — Supplementary Information [file 41467_2024_51993_MOESM1_ESM.pdf]

Supplementary Information for:
A bitopic agonist bound to the dopamine 3 receptor reveals a selectivity site

Sandra Arroyo-Urea[1,2], Antonina L. Nazarova[3,4], Ángela Carrión-Antolí[1,2], Alessandro Bonifazi[5], Francisco O. Battiti[5], Jordy Homing Lam[3,4], Amy Hauck Newman[5], Vsevolod Katritch[3,4,6] and Javier García-Nafría[1]*

[1]Institute for Biocomputation and Physics of Complex Systems (BIFI), University of Zaragoza, 50018, Zaragoza, Spain

[2]Laboratory of Advanced Microscopy (LMA), University of Zaragoza, 50018, Zaragoza, Spain.

[3]Department of Quantitative and Computational Biology, University of Southern California, Los Angeles, CA 90089, USA

[4]Center for New Technologies in Drug Discovery and Development, Bridge Institute, Michelson Center for Convergent Biosciences, University of Southern California, Los Angeles, CA, USA.

[5]Medicinal Chemistry Section, Molecular Targets and Medications Discovery Branch, National Institute on Drug Abuse – Intramural Research Program, National Institutes of Health, 333 Cassell Drive, Baltimore, Maryland 21224, USA

[6]Department of Chemistry, University of Southern California, Los Angeles, CA, USA.

* Corresponding author:
Javier García-Nafría
Email: jgarcianafria@unizar.es

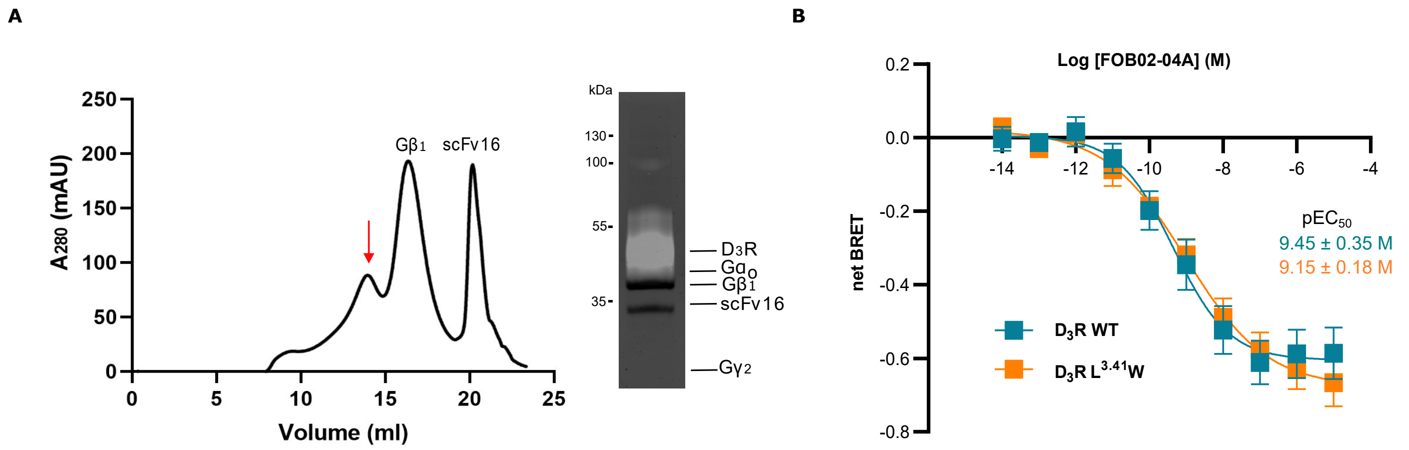

Supplementary Figure 1. Purification of the D3R:Go:FOB02-04A complex and validation of the $L^{3.41}W$ mutation. A Size exclusion chromatogram of the D3R:G_O:FOB02-04A sample (left). SDS-PAGE of the pure D3R:G_O:FOB02-04A sample with superposed in-gel fluorescence for the GFP-D3R (right). **B** Concentration-response curves of D3R WT (blue) compared to $L^{3.41}W$ variant (orange) upon G_{OA} activation with FOB02-04A using the TRUPATH assay. Data are presented as means ± SEM of five (D_3R WT) and six ($L^{3.41}W$) independent experiments performed in technical triplicate. Source data are provided as a Source Data file.

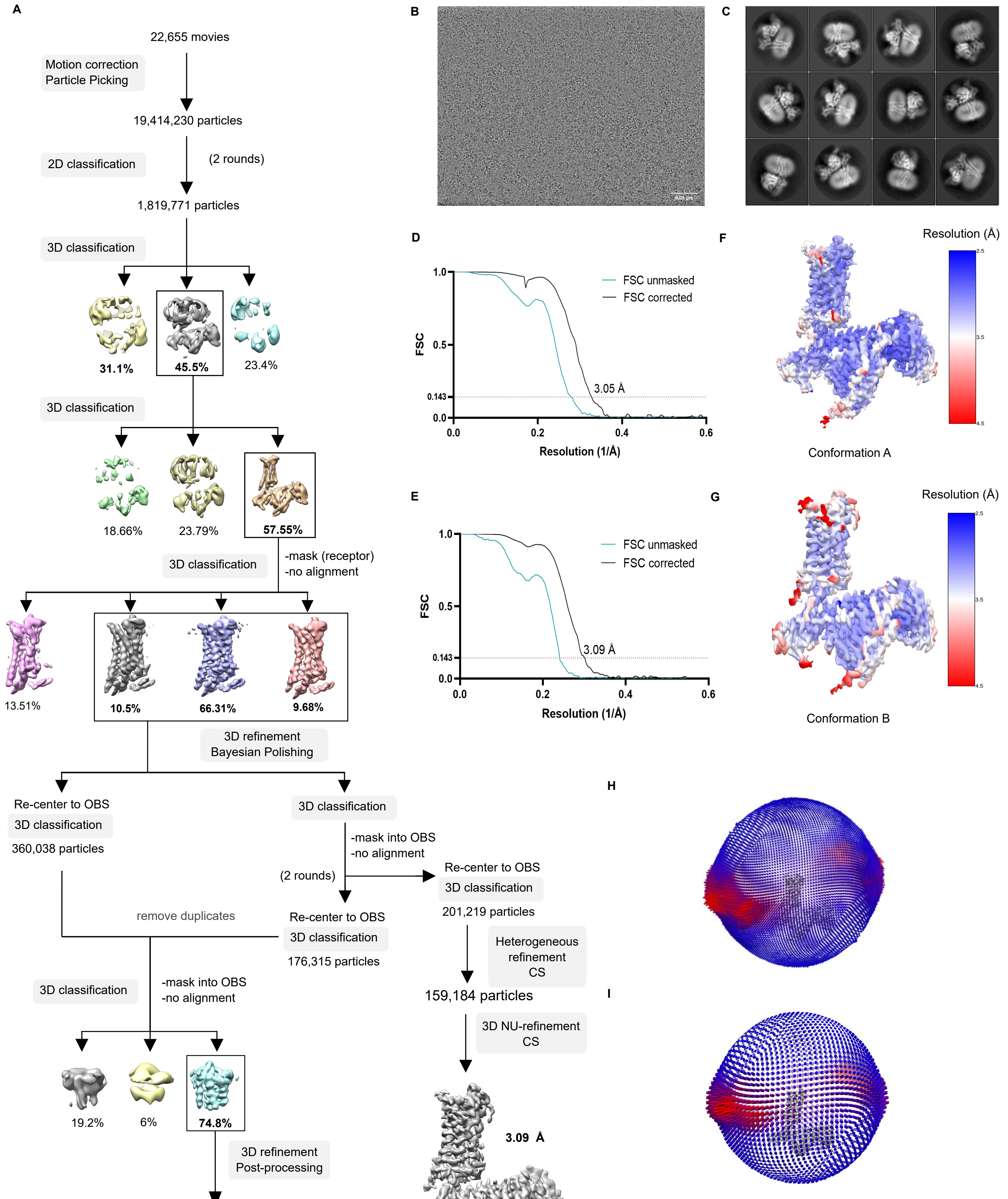

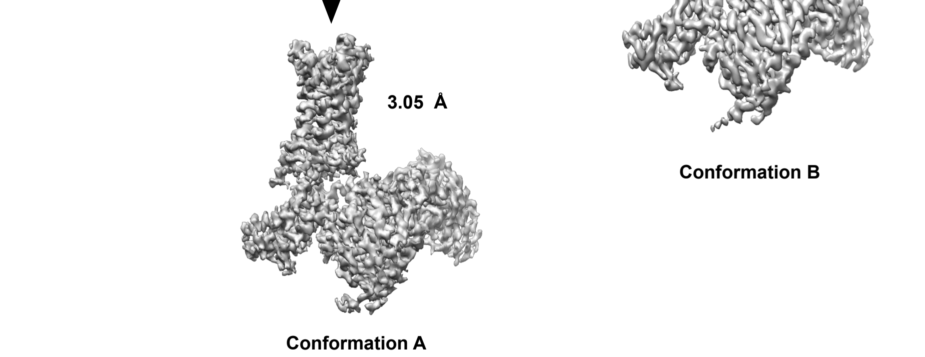

Supplementary Figure 2. Cryo-EM of the single particle reconstruction of the D$_3$R:G$_O$:FOB02-04A complex structure. A Flow chart of data processing. **B** Representative micrograph (0.84 Å/ pixel) of the D$_3$R:G$_O$:FOB02-04A dataset collected using a Titan Krios with a K3 detector. **C** Representative 2D class averages of the D$_3$R:G$_O$:FOB02-04A complex. **D-E** FSC curve of the final reconstruction showing an overall resolution of 3.05 Å and 3.09 Å for Conformation A **(D)** and B **(E)**. **F-G** Local resolution estimation of the D$_3$R:G$_O$:FOB02-04A map as calculated by CryoSPARC for Conformation A **(F)** and Conformation B **(G)**. **H-I** Angular distribution of all particles used in the final reconstruction for Conformation A **(H)** and Conformation B **(I)**.

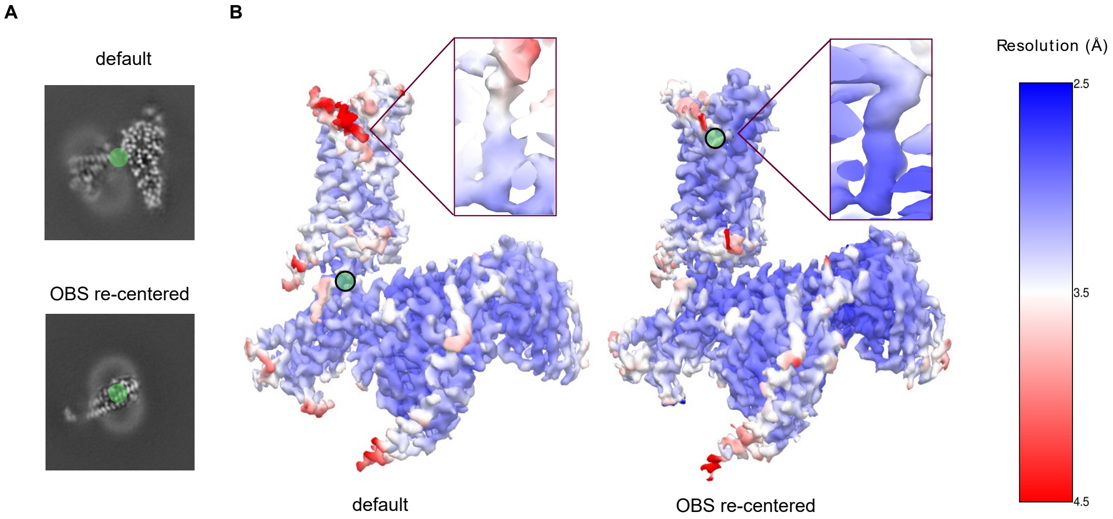

Supplementary Figure 3. Effect of centering the ligand binding site on the cryo-EM box. A Slices from 3D refinement before and after re-centering the particles at the OBS. **B** Local resolution of the cryo-EM maps (as calculated by CryoSPARC) with an inset into the ligand binding site before and after OBS re-centering. The center of the box is marked with a green dot in the 2D slices and 3D maps.

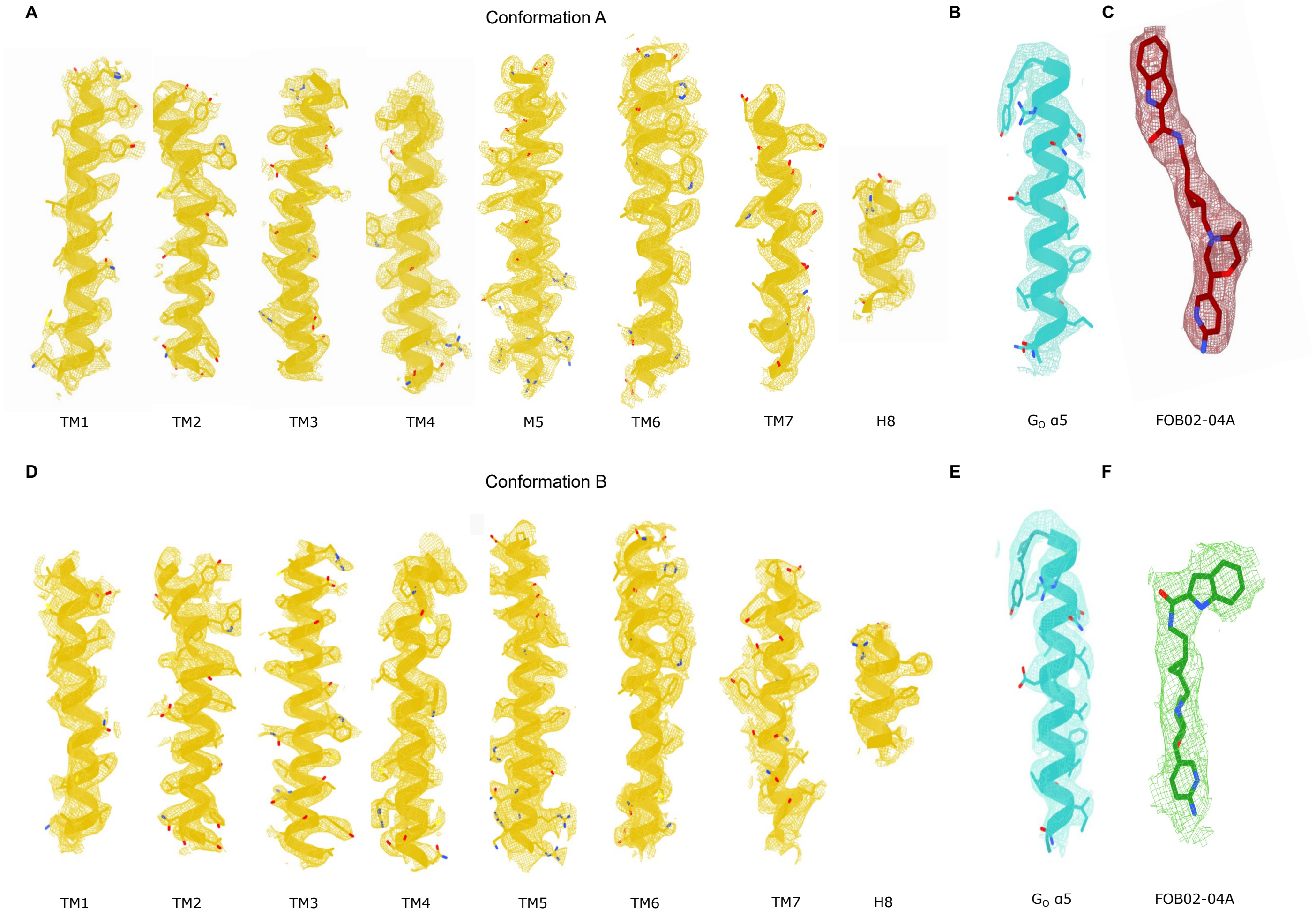

Supplementary Figure 4. Cryo-EM map quality and model fit of the D3R:G_O:FOB02-04A complexes. Protein shown as cartoons with residues represented as sticks and colored by subunit (yellow D3R and cyan Gα_O) or ligand (dark red for Conformation A and green for Conformation B). Cryo-EM density is shown as mesh with color corresponding to each component. **A-C** Transmembrane helices of D3R Conformation A, the α5-helix of G_O and FOB02-04A in Conformation A. **D-F** Transmembrane helices of D3R Conformation B, the α5-helix of G_O and FOB02-04A in Conformation B.

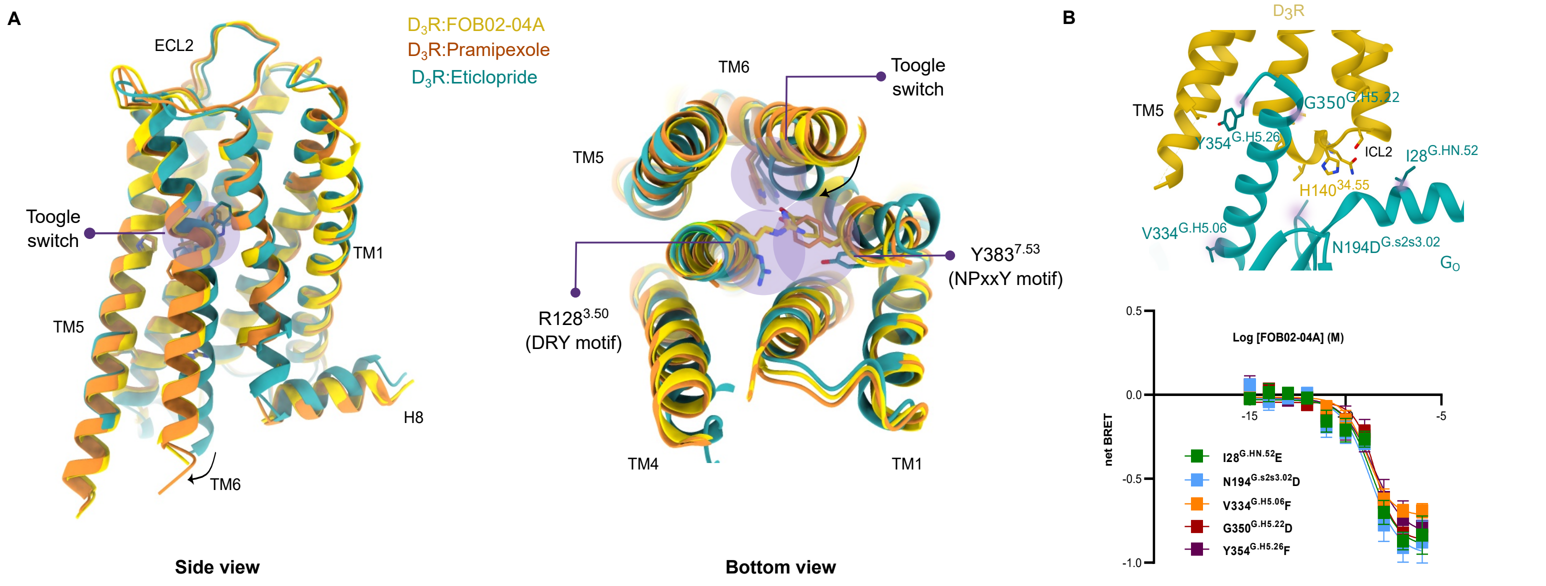

Supplementary Figure 5. Activation of the D_3R by FOB02-04A. A Two orthogonal views of the D_3R-FOB02-04A (yellow, cartoons) superposed to the inactive state eticlopride-bound (PDB 3PBL, cyan cartoons) and active state pramipexole-bound D_3R (PDB 7CMU, orange cartoons). Conserved motifs are highlighted with relevant residues displayed as sticks. **B** Overall view of G_o selected mutations to G_i equivalent residues (highlighted in violet) (top panel) and concentration-response curves of D_3R upon G_{OA} mutants activation by FOB02-04A using TRUPATH assay (shown as net BRET, bottom panel). Data are derived from three (G_{OA} I28$^{G.HN.52}$E, N194$^{G.s2s3.02}$D, Y354$^{G.H5.26}$F) and four (G_{OA}-V334$^{G.H5.06}$F, G350$^{G.H5.22}$D) independent experiments performed in technical triplicate.Source data are provided as a Source Data File.

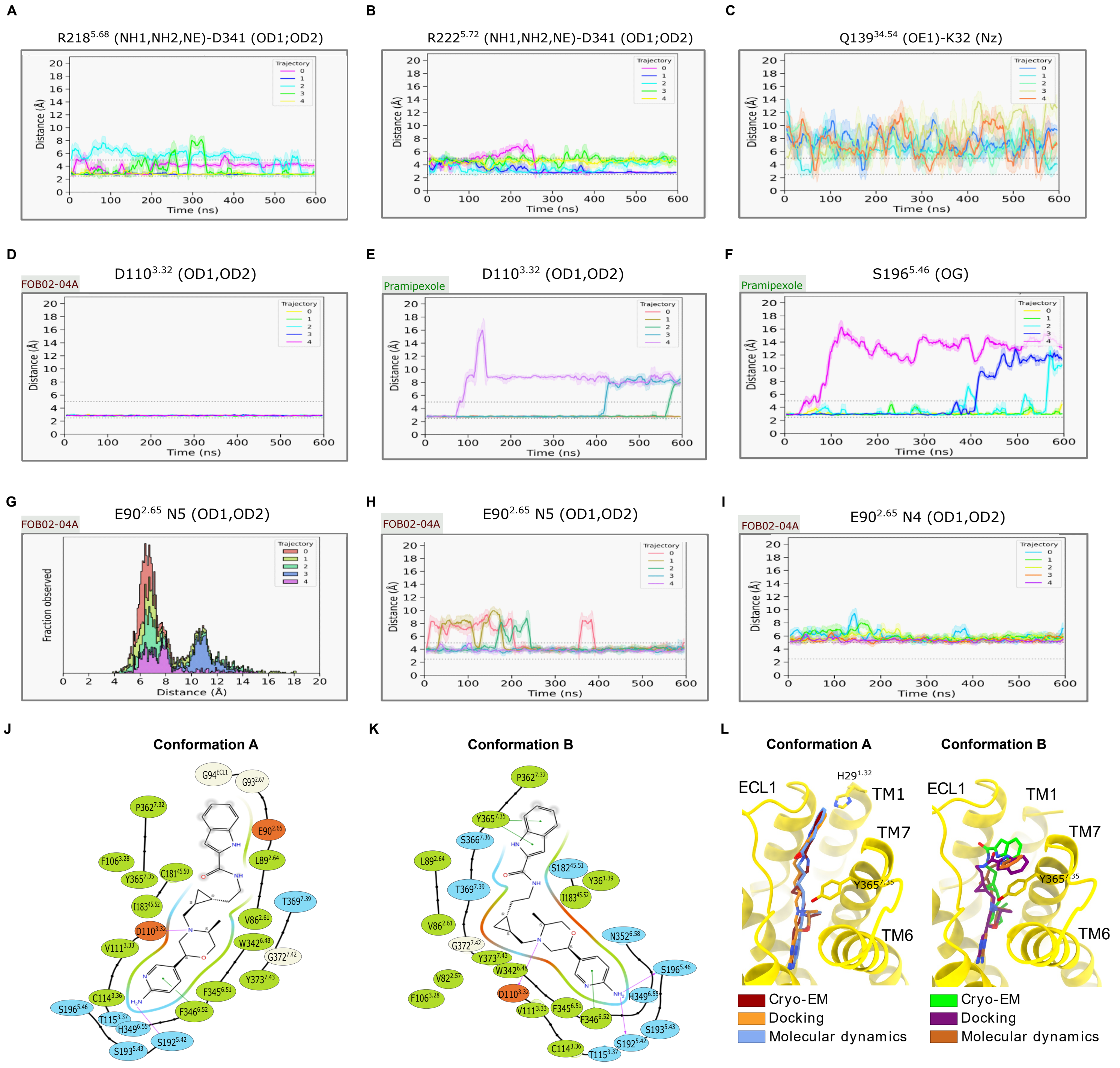

Supplementary Figure 6. MD simulations of D$_3$R:G$\alpha_O\beta\gamma$ interactions with bitopic FOB02-04A and pramipexole. A-B Salt bridge interaction between negatively charged carboxyl group of polar residue D341$^{G.H5.13}$ of Gα_O C-terminal α5 and positively charged guanidinium group of FOB02-04A:D$_3$R polar residue R218$^{5.68}$ **(A)** and polar residue R222$^{5.72}$ **(B)**. **C** Closest distance between Q139$^{34.54}$ in D$_3$R and terminal tertiary amine moeiety of K32$^{G.hsn1.03}$ of Gα_O N-terminal helix. **D-E** Salt bridge interaction between oxygen atoms of carboxyl group of D110$^{3.32}$ in D$_3$R with the basic nitrogen of trans-cyclopropyl amine linkage of FOB02-04A **(D)** and with the atom N1 of the propylamino group of pramipexole **(E)**. **F** Hydrogen bond interaction between oxygen atom S196$^{5.46}$ in D$_3$R with the atom type bitopic FOB0204-A within the D$_3$R:G$\alpha_O\beta\gamma$ complex. **G** Frequency of interactions indicates the presence of two distinct conformational states of estimated distribution of 90% for Conformation A and 10% for Conformation B. **H-I** Closest distances between oxygen atoms of carboxyl group of E90$^{2.65}$ in D$_3$R with the atom type N5 of the indole moiety **(H)** and atom N4 of the amide group of FOB02-04A **(I)**. Data from five independent simulations of D$_3$R:G$\alpha_O\beta\gamma$ heterotrimer complex are shown, spanning 0.6 μs of cumulative time per system, with the sampling rate of 10 frames per ns, solid lines and same-color bonding interactions are shown at 5.0 Å and 2.5 Å thresholds (grey, dashed lines). **J-K** 2D interaction diagram between D$_3$R with D$_3$R:G$\alpha_O\beta\gamma$ and ligands **(J)** bitopic FOB02-04A Conformation A and **(K)** bitopic FOB02-04A Conformation B. Specific residues in the binding pocket that interact are shown as sticks and are labelled. Color code for residues and interactions: green, hydrophobic; blue, polar; red, negatively charged; grey, glycine. The solid purple arrow line shows the H-bonding interaction, solid green line shows the π-π-π stacking interaction. **L** Comparison of FOB02-04A poses obtained from cryo-EM structure with those predicted by molecular docking and molecular dynamic simulations for Conformation A (left panel) and B (right panel).

A

FOB02-04A D3R	Potencies pEC$_{50}$ (M) ± SEM	Δ	Efficacies (% of WT) ± SEM	Δ	Expression (% of WT) ± SEM
WT	9.45 ± 0.35	0	100 ± 11.25	0	100
L119$^{3.41}$W	9.15 ± 0.18	-0.3	108.12 ± 9.50	8.12	170.6 ± 11.9
H29$^{1.32}$A	8.94 ± 0.45	-0.51	54.72 ± 12.05	-45.28	113 ± 2.4
H29$^{1.32}$F	9.59 ± 0.05	0.14	40.86 ± 5.47	-59.14	69.9 ± 6.5
H29$^{1.32}$K	9.44 ± 0.08	-0.01	75.82 ± 4.88	-24.18	88 ± 17.0
H29$^{1.32}$R	9.18 ± 0.15	-0.27	50.4 ± 3.88	-49.6	82.39 ± 7.3
V86$^{2.61}$A	9.99 ± 0.21	0.54	65.16 ± 9.29	-34.84	72.5 ± 1.0
L89$^{2.64}$A	8.74 ± 0.27	-0.71	83.86 ± 14.49	-16.14	95.3 ± 13.7
E90$^{2.65}$A	9.57 ± 0.29	0.12	105.67 ± 6.56	5.67	154.3 ± 3.1
ΔG94^{ECL1}	ND	ND	ND	ND	130 ± 15.5
F106$^{3.28}$A	8.57 ± 0.48	-0.88	50.12 ± 3.81	-49.88	121.1 ± 7.4
D110$^{3.32}$A	ND	ND	ND	ND	79.7 ± 13.3
V111$^{3.33}$A	7.39 ± 0.38	-2.06	94.72 ± 6.53	-5.26	63 ± 9.4
T115$^{3.37}$A	7.66 ± 0.41	-1.79	91.19 ± 15.14	-8.81	88.9 ± 9.9
I183$^{45.52}$A	7.17 ± 0.11	-2.28	100.85 3.84	0.85	79.7 ± 5.2
S192$^{5.42}$A	10.02 ± 0.33	0.57	78.39 ± 14.71	-21.61	106.2 ± 6.4
S193$^{5.43}$A	10.39 ± 0.42	0.94	98.15 ± 30.65	-1.85	207.3 ± 9.1
S196$^{5.46}$A	9.24 ± 0.83	-0.21	63.37 ± 13.13	-36.53	238.1 ± 17.5
W342$^{6.48}$A	ND	ND	ND	ND	100.9 ± 4.3
F345$^{6.51}$A	10.03 ± 0.3	0.58	118.44 ± 19.86	18.44	183 ± 9.9
F346$^{6.52}$A	10.00 ± 0.29	0.55	69.55 ± 4	-30.41	126.8 ± 2
Y365$^{7.35}$A	9.64 ± 0.49	0.19	112.43 ± 10.83	12.43	105 ± 2.4
T369$^{7.39}$A	10.89 ± 0.33	1.44	90.11 ± 9.42	-9.89	125.5 ± 0.8
Y373$^{7.43}$A	ND	ND	ND	ND	110 ± 8.0
D2R	7.48 ± 0.21	-1.97	110.96 ± 6.43	10.96	215.3 ± 18
D2R Y408$^{7.35}$A	8.11 ± 0.39	-1.34	131.8 ± 7.06	31.8	155.6 ± 17.3

B

FOB02-04A D3R-Go mutants	Potencies pEC$_{50}$ (M) ± SEM	Δ	Efficacies (% of WT) ± SEM	Δ
I28$^{G.HN.52}$E	9.06± 0.39	-0.39	161.65 ± 10.7	61.65
N194$^{G.s2s3.02}$D	8.69 ± 0.15	-0.76	169.18 ± 13.07	69.18
V334$^{G.H5.06}$F	8.84 ± 0.09	-0.61	130.8 ± 5.01	30.8
G350$^{G.H5.22}$D	8.43 ± 0.09	-1.02	158.0 ± 4.31	58
Y354$^{G.H5.26}$F	8.90 ± 0.35	-0.55	144.5 ± 9.87	44.5
Gi WT	8.17 ± 0.59	-1.28	98.9 ± 14.83	-1.1

C

Pramipexole D3R	Potencies pEC$_{50}$ (M) ± SEM	Δ	Efficacies (% of WT) ± SEM	Δ
WT	9.32 ± 0.5	0	95.4.0 ± 27	-4.6
H29$^{1.32}$A	9.93 ± 0.56	0.61	54.5 ± 17.9	-40.9
H29$^{1.32}$F	9.21 ± 0.21	0.11	89.49 ± 14.25	5.91

Quinpirole D3R	Potencies pEC$_{50}$ (M) ± SEM	Δ	Efficacies (% of WT) ± SEM	Δ
WT	8.90 ± 0.049	0	97.48 ± 2.28	0
H29$^{1.32}$A	8.28 ± 0.36	-0.615	72.6± 12.0	-24.88
ΔG94^{ECL1}	9.79 ± 0.60	0.89	82.24 ± 23	-15.24

D

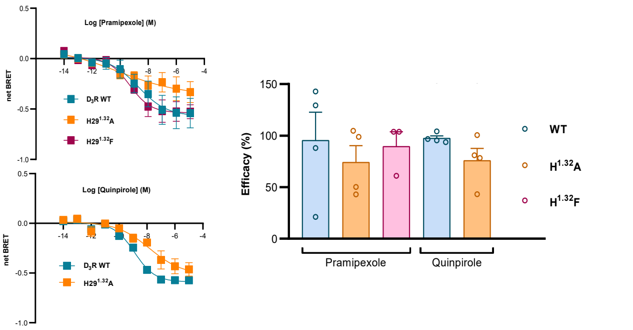

E

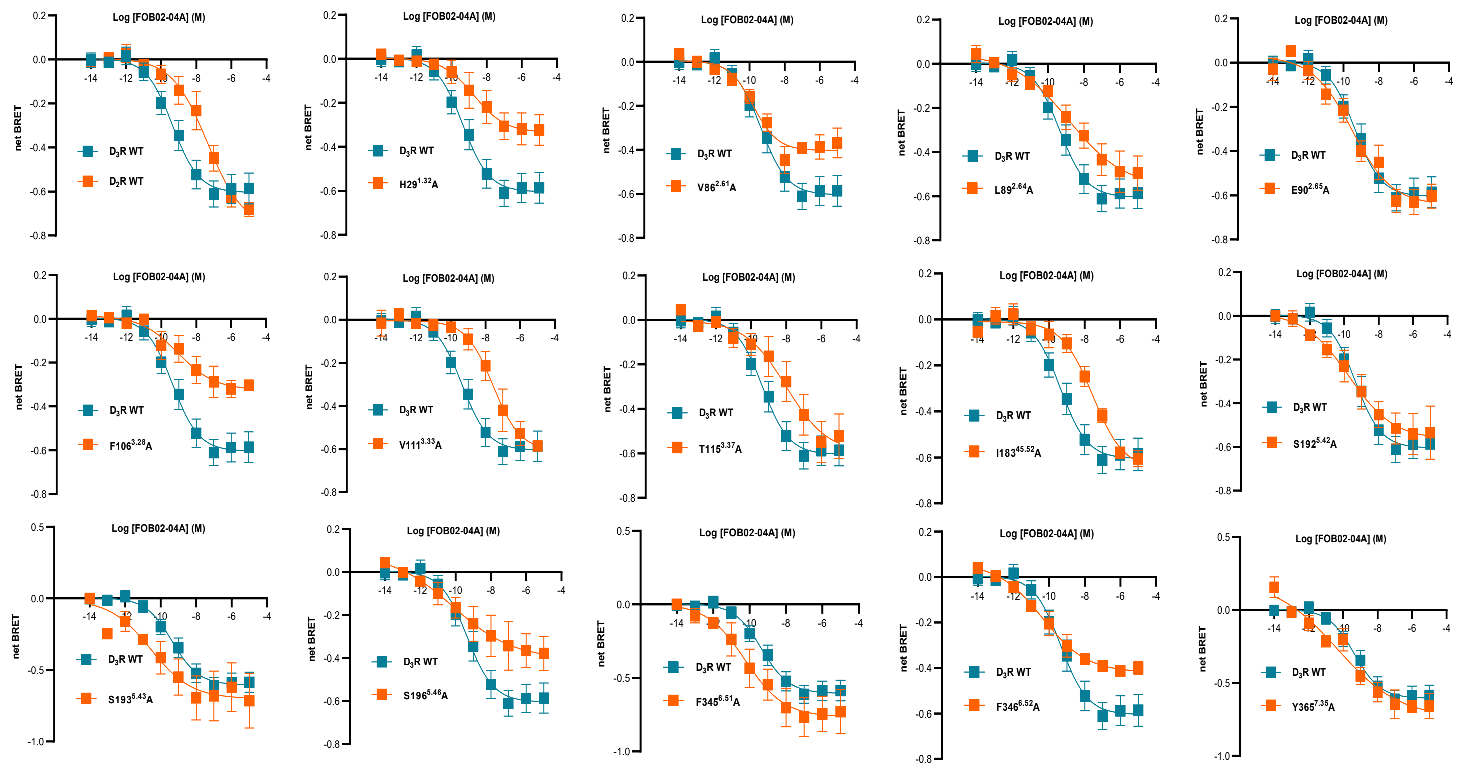

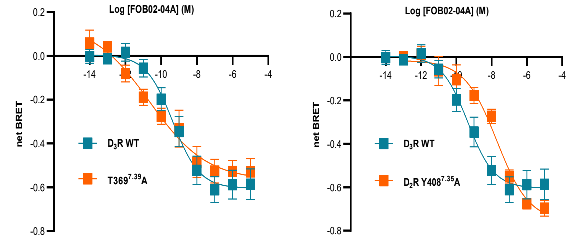

Supplementary Figure 7. Comparisons of activity and expression of WT mutant D$_3$R and G$_O$ variants. A-C Tables with summary of pEC$_{50}$ values, Emax and expression levels. pEC$_{50}$ values and Emax of all data are derived from four independent experiments performed in technical triplicates (n=4) except for D110$^{3.32}$A,S196$^{5.46}$A,Y365$^{7.35}$A,T369$^{7.39}$A,W342$^{6.48}$A,Y373$^{7.43}$A,G$_{OA}$ I28$^{G.HN.52}$E, N194$^{G.s2s3.02}$D, Y354$^{G.H5.26}$F H29$^{1.32}$F,H29$^{1.32}$K,H29$^{1.32}$R(n=3), WT, V86$^{2.61}$A,L89$^{2.64}$A,E90$^{2.65}$A,ΔG94^{ECL1},F346$^{6.52}$A (n=5) and L119$^{3.41}$W, T115$^{3.37}$A (n=6).The expression levels of mutant D$_3$R were normalized to WT D$_3$R as 100% and are derived from three independent experiments performed in technical triplicates (n=3) except for D$_2$R (n=4), L89$^{2.64}$A, V111$^{3.33}$A (n=5) and T115$^{3.37}$A (n=6). Colors are based on the effects of mutations on receptor activity with orange for reduced activity and green for increased activity. Definitions: ND-not detectable; Δ-calculated difference of mutant from WT. **D** Concentration-response curves and Emax values for H29$^{1.32}$ mutants upon activation of G$_{OA}$ using TRUPATH assays in the presence of pramipexole or quinpirole. Emax values have been normalized to D$_3$R WT activity in the presence of FOB02-04A and are derived from four independent experiments performed in technical triplicates (n=4) except for H29$^{1.32}$F (n=3) (Holm-Sidak multiple comparisons tests two tailed p value). **E** Concentration-response curves for WT (blue) and mutant D$_3$R (orange) following FOB02-04A G$_{OA}$ activation obtained using the TRUPATH assay (shown as net BRET). Data are presented as means ± SEM derived from four independent experiments performed in technical triplicates (n=4) except for S196$^{5.46}$A,Y365$^{7.35}$A,T369$^{7.39}$A (n=3), WT, V86$^{2.61}$A,L89$^{2.64}$A,E90$^{2.65}$A,F346$^{6.52}$A (n=5) and T115$^{3.37}$A (n=6). All functional source data are provided as a source data file.

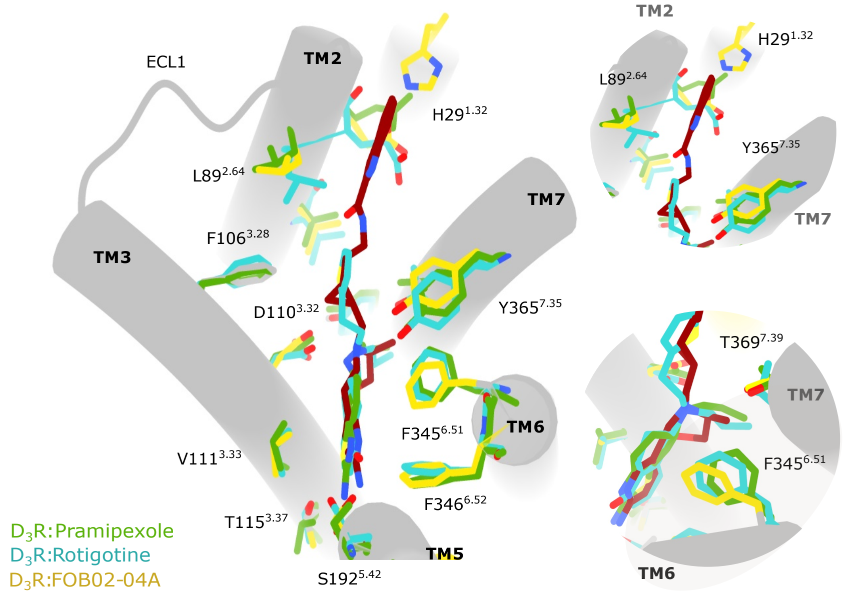

Supplementary Figure 8. Comparisons of FOB02-04A, pramipexole and rotigotine binding site on the D_3R. FOB02-04A, pramipexole and rotigotine are depicted as red, green and cyan sticks. D_3R is shown as grey cartoons with relevant residues as sticks with carbon colour corresponding to the agonist (red, green and cyan for FOB02-04A, pramipexole and rotigotine respectively).

FOB02-04A

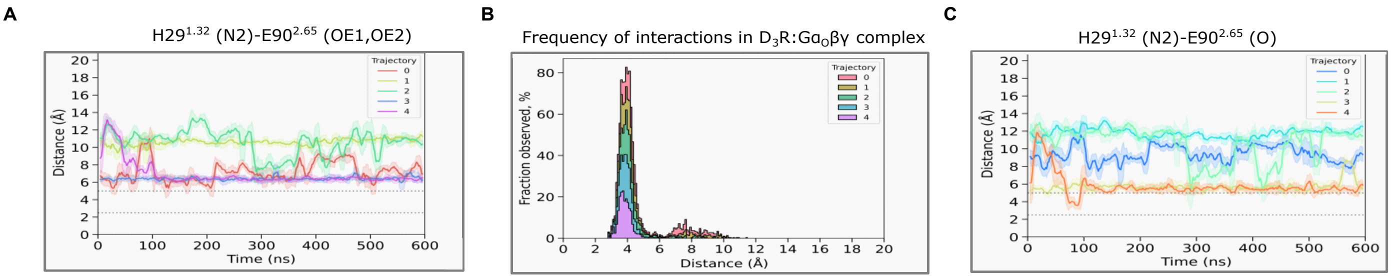

Pramipexole

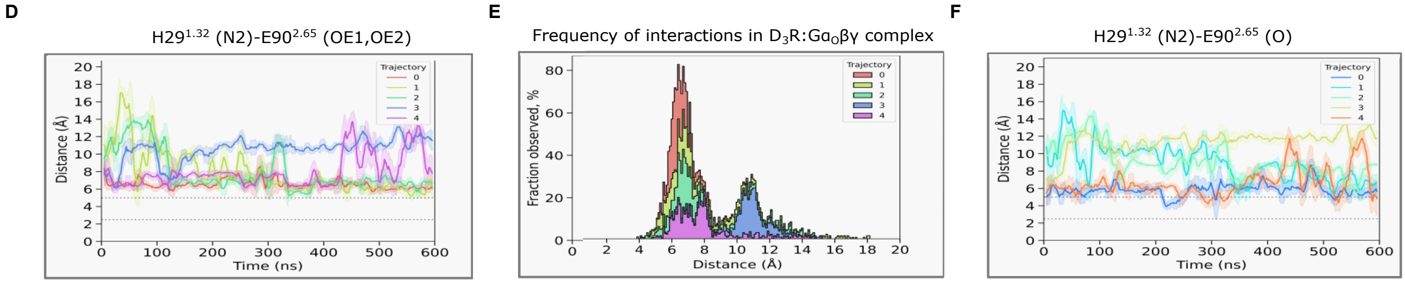

Supplementary Figure 9. MD analysis of TM1 stability in interactions of D$_3$R:G$\alpha_O\beta\gamma$ with bitopic FOB02-04A (A-C) and pramipexole (D-F). A Closest distances between the carboxyl group of E90$^{2.65}$ and the protonated N(ε) of H29$^{1.32}$ with FOB02-04A. **B** Frequency of interactions between the same groups in the presence of FOB02-04A. **C** Closest distances between backbone oxygen atom of E90$^{2.65}$ and the protonated N(ε) of H29$^{1.32}$ with FOB02-04A. **D** Closest distances between the carboxyl group of E90$^{2.65}$ and the protonated N(ε) of H29$^{1.32}$ with pramipexole (PDB 7CMU). **E** Frequency of interactions between the same groups in the presence of pramipexole (PDB 7CMU). **F** Closest distances between backbone oxygen atom of E90$^{2.65}$ and the protonated N(ε) of H29$^{1.32}$ with pramipexole (PDB 7CMU). Five independent simulations of D$_3$R-G$\alpha_O\beta\gamma$ heterotrimer complex are shown, spanning 0.6 μs of cumulative time per system, with the sampling rate of 10 frames per ns, solid lines and same-color shadows representing moving average values and one standard deviation respectively from 50 frames in all cases. Upper and lower boundaries for hydrogen bonding interactions are shown at 5.0 Å and 2.5 Å thresholds (grey, dashed lines).

Supplementary Table 1. Cryo-EM data collection, refinement and validation statistics.

	D_3R:G$\alpha_O\beta\gamma$:FOB02-04A Conformation A	D_3R:G$\alpha_O\beta\gamma$:FOB02-04A Conformation B
**Data collection and processing**		
Microscope	FEI Titan Krios	FEI Titan Krios
ESRF data identification	10.15151/ESRF-ES-751565769	10.15151/ESRF-ES-751565769
Detector	K3 + GIF	K3 + GIF
Magnification	105,000x	105,000x
Voltage (kV)	300	300
Electron exposure (e–/Å^2)	49.88662	49.88662
Defocus range (μm)	-1 to -3	-1 to -3
Pixel size (Å)	0.84	0.84
Symmetry imposed	C1	C1
Micrographs	22,655	22,655
Final particle images (no.)	275,383	159,184
Map resolution (Å)	3.05	3.09
FSC threshold	0.143	0.143
**Refinement**		
Initial model used (PDB code)	7CMV, 6K41	7CMV, 6K41
Model resolution[1] (Å)	3.1	3.4
FSC threshold	0.5	0.5
Map sharpening *B* factor (Å^2)	-100	-156.9
Model composition		
Non-hydrogen atoms	8,666	8,633
Protein residues	1,115	1,112
Ligands		
B factors (Å^2)		
Protein	78.52	118.04
Ligand	48.81	106.34
R.m.s. deviations		
Bond lengths (Å)	0.003	0.01
Bond angles (°)	0.51	1.06
Validation		
MolProbity score	1.33	1.30
Clashscore	4.89	5.61
Poor rotamers (%)	0.21	0.53
EMRinger score	3.22	2.67
Ramachandran plot		
Favored (%)	95.44	98.35
Allowed (%)	4.47	1.55
Disallowed (%)	0.09	0.09
PDB/EMDB codes	9F33/50168	9F34/50169

[1]Resolution at which FSC between map and model is 0.5

Supplementary Table 2. Molecular Dynamics Summary Table

**Reliability and reproducibility checklist for molecular dynamics simulations**<br>***All boxes must be marked YES by acceptance unless an N/A option is available**	**Yes**	**N/A**	**Response (Please state where this information can be found in the text)**
**1. Convergence of simulations and analysis**			
1a. Is an evaluation presented in the text to show that the property being measured has equilibrated in the simulations (*e.g.* time-course analysis)?	☒		The information can be found in the Molecular Dynamics Simulations section within Methods
1b. Then, is it described in the text how simulations are split into equilibration and production runs and how much data were analyzed from production runs?	☒		The information can be found in the Molecular Dynamics Simulations section within Methods
1c. Are there at least 3 simulations per simulation condition with statistical analysis?	☒		The information can be found in Supplementary Information, Fig.6 as well Activation mechanism and GO coupling of the D3R bound to FOB02-04A section of the manuscript.
1d. Is evidence provided in the text that the simulation results presented are independent of initial configuration?	☒		The information can be found in the Molecular Dynamics Simulations section of the Methods section of the manuscript
**2. Connection to experiments**			
2a. Are calculations provided that can connect to experiments (*e.g.* loss or gain in function from mutagenesis, binding assays, NMR chemical shifts, J-couplings, SAXS curves, interaction distances or FRET distances, structure factors, diffusion coefficients, bulk modulus and other mechanical properties, *etc.*)?	☒		The MD results are thoroughly connected to Cryo-EM structural information as well as mutational and functional results.
**3. Method choice**			
3a. Is it described in the text what force field and water model are used and why?	☒		The information can be found in the Molecular Dynamics Simulations section of the Methods section of the manuscript
3b. Do simulations contain membranes, membrane proteins, intrinsically disordered proteins, glycans, nucleic acids, polymers, or cryptic ligand binding?	☒	☐	The information can be found in the Molecular Dynamics Simulations section of the Methods section of the manuscript
If 3b is **YES**, are enhanced sampling methods used?	☐	☒	Response not needed if **N/A**
If enhanced sampling methods are used, are the convergence criteria clearly stated?	☐		
If 3b is **YES**, is it explained in the text why or why not enhanced sampling methods are used?	☒		We have added the following to the Molecular

				Dynamics Simulations section of the Methods: *Since the structural insights into the binding mode of the D_3 receptor bound to a bitopic agonist were efficiently achieved using standard MD simulations, without the need to explore rare events or surmount significant energy barriers, no enhanced sampling methods were required.*
**4. Code and reproducibility**				
4a. Is a table provided describing the system setup, such as simulation box dimensions, total number of atoms, total number of water molecules, salt concentration, lipid composition (number of molecules and type)?		☒		The information can be found in the Molecular Dynamics Simulations section of the Methods section. An additional Supplementary table has been added to describe the system composition.
4b. Is it described in the text what simulation and analysis software and which versions are used?		☒		The information can be found in the Molecular Dynamics Simulations section of the Methods section of the manuscript
4c. Are initial coordinate and simulation input files and a coordinate file of the final output provided as supplementary files or in a public repository?		☒		The information can be found in the Data availability section of the manuscript. The trajectories for the Molecular Dynamics simulations have been deposited as an open-access Zenodo repository.
4d. Is there custom code or custom force field parameters?		☐	☒	Response not needed if **N/A**
	If **YES**, are they provided as supplementary profiles or in a public repository?	☐		

Supplementary Table 3. Number of atoms in the assembled system for Molecular Dynamics simulations.

Composition	D_3R:Gαβγ:FOB02-04A (molecules/ atoms)	D_3R:Gαβγ:Pramipexol (molecules/atoms)
**D_3 receptor**	1/4427	1/4427
**FOB02-04A**	1/64	1/33
**$G\alpha_O$ protein**	1/5545	1/5545
**Gβ protein**	1/5094	1/5094
**Gγ protein**	1/889	1/911
**Cholesterol (CHL1)**	120/8880	120/8880
**DPPC**	220/28600	220/28600
**DOPC**	60/8280	60/8280
**Sodium**	112	108
**Chloride**	104	102
**Water**	38818/115722	37934/113802
