# Supplementary material for: A bitopic agonist bound to the dopamine 3 receptor reveals a selectivity site
Source: Nat Commun. 2024 Sep 5;15:7759. doi: 10.1038/s41467-024-51993-4 (PMC11377762; doi:10.1038/s41467-024-51993-4)
Supplement: Supplementary file 3 — Description of Additional Supplementary Files [file 41467_2024_51993_MOESM3_ESM.pdf]

File name: Supplementary Data 1
Description: Sequences of oligos used in molecular cloning during this work.

Oligo name	Sequence
**$G_{O/i}$ mutations**	
Go_i_I28E_Fw	aggatggcgagagcgccgccaaagac
Go_i_I28E_Rv	gcggcgctctcgccatcctctttgaggttttt
Go_i_N194D_Fw	cattcaaggatctccacttcaggctgtttgac
Go_i_N194D_Rv	gaagtggagatccttgaatgtgaagtgggtt
Go_i_V334F_Fw	acatccagtttgtgttcgacgccgtcac
Go_i_V334F_Rv	tcgaacacaaactggatgttattcgtgtctgtg
Go_I_G350D_Fw	acctccgggattgcggcttgtactgat
Go_i_G350D_Rv	aagccgcaatcccggaggttgttggc
Go_i_Y354F_Fw	gcggcttgttttgatagctcgagtctagag
Go_i_Y354F_Rv	agctatcaaaacaagccgcagccccg

**D_3R alanine mutations**	
D3_V86A_Fw	cttggtgatgccctgggccgtatacctggaggtgacagg
D3_L89A_Fw	cttggtgatgccctgggtggtatacgccgaggtgac
D3_E90A_Fw	cttggtgatgccctgggtggtatacctggccgtgacagg
D3_Rv_85_94	cccagggcatcaccaaggtggccaccagc
D3_S192A_Fw	caaccctgattttgtcatctacgcctcagtggtgtc
D3_S193A_Fw	caaccctgattttgtcatctactctgccgtggtgtc
D3_S196A_Fw	caaccctgattttgtcatctactcttcagtggtggccttctacct
D3_Rv_192_196	gtagatgacaaaatcagggttggagatggagcag
D3_F106A_Fw	gccgcatttgctgtgatgttgccgtcaccctgg
D3_D110A_Fw	gccgcatttgctgtgatgtttttgtcaccctggccgtcatga
D3_V111A_Fw	gccgcatttgctgtgatgtttttgtcaccctggatgccatgatg
D3_Rv_106_111	aacatcacagcaaatgcggctgaaattc
D3_S182A_Fw	ggggaccccactgtctgcgccatctcc
D3_I183A_Fw	ggggaccccactgtctgctccgcctccaacc
D3_Rv_182_183	gacagtggggtcccctgtggtattaaag
D3_F345A_Fw	tgtctgctggctgcccgccttcttgaccc
D3_F346A_Fw	tgtctgctggctgcccttcgccttgaccc
D3_Rv_345_346	gggcagccagcagacaatg
D3_T368A_Fw	ccagagctttacagtgccgccacatggctgg
D3_W369A_Fw	ccagagctttacagtgccacgacagccctgggctacgtg
D3_Rv_368_372	ggcactgtaaagctctggggacacgtggcatg
D3_P362A_Fw	cagacatgccacgtgtccgccgagctttacag
D3_Y365A_Fw	cagacatgccacgtgtccccagagcttgccagtgccac
D3_Rv_362_365	ggacacgtggcatgtctggcagtg
D3_deltaG94_Fw	gtatacctggaggtgacaggtgtctggaatttcagccgc
D3_deltaG94_Rv	acctgtcacctccaggtataccac
D3_H29A_Fw	cagccaggcccgcccagccgcctactatgccctctcctac
D3_H29_Rv	ggctgggcgggcctggctggcacc
D3_Y373A_Fw	ccagagctttacagtgccacgacatggctgggcgccgtgaatag
D3_Rv_368_373	ggcactgtaaagctctggggacacgtggcatg
D3_T115A_Rv	tgacatccagggtgacaaaaacatcacagcaaatgcggct

D3_T115A_Fw	tttttgtcaccctggatgtcatgatgtgtgccgccagcat

D3_W342A_Fw	tgtgcttggggccttcattgtctgcgccctgcccttcttc
D3_W342A_Rv	atgaaggccccaagcacaatggccaccatttg
**D_2R alanine mutants**	
D2_Y408A_Fw	gtgattgcaacattccaccagtgctcgccagcgcctttac
D2_Y408A_Rv	ctggtggaatgttgcaatcacaatgaatgttcaggatgtg
**Additional D_3R $H29^{1.32}$ mutants**	
H29R_Fw	cagccaggcccgcccaagagcctactatgccctctcctac
H29F_Fw	cagccaggcccgcccattcgcctactatgccctctcctac
H29K_Fw	cagccaggcccgcccaaaggcctactatgccctctcctac
H29_Rv	tgggcgggcctggctggcacctgtgga
