# Supplementary material for: A bitopic agonist bound to the dopamine 3 receptor reveals a selectivity site
Source: Nat Commun. 2024 Sep 5;15:7759. doi: 10.1038/s41467-024-51993-4 (PMC11377762; doi:10.1038/s41467-024-51993-4)
Supplement: Supplementary file 4 — Reporting Summary [file 41467_2024_51993_MOESM4_ESM.pdf]

nature portfolio

Corresponding author(s): Javier García Nafría

Last updated by author(s): 15/7/24

Reporting Summary

Nature Portfolio wishes to improve the reproducibility of the work that we publish. This form provides structure for consistency and transparency in reporting. For further information on Nature Portfolio policies, see our Editorial Policies and the Editorial Policy Checklist.

Statistics

For all statistical analyses, confirm that the following items are present in the figure legend, table legend, main text, or Methods section.

n/a	Confirmed	
☐	☒	The exact sample size (*n*) for each experimental group/condition, given as a discrete number and unit of measurement
☐	☒	A statement on whether measurements were taken from distinct samples or whether the same sample was measured repeatedly
☐	☒	The statistical test(s) used AND whether they are one- or two-sided<br>*Only common tests should be described solely by name; describe more complex techniques in the Methods section.*
☒	☐	A description of all covariates tested
☒	☐	A description of any assumptions or corrections, such as tests of normality and adjustment for multiple comparisons
☐	☒	A full description of the statistical parameters including central tendency (e.g. means) or other basic estimates (e.g. regression coefficient) AND variation (e.g. standard deviation) or associated estimates of uncertainty (e.g. confidence intervals)
☐	☒	For null hypothesis testing, the test statistic (e.g. *F*, *t*, *r*) with confidence intervals, effect sizes, degrees of freedom and *P* value noted<br>*Give P values as exact values whenever suitable.*
☒	☐	For Bayesian analysis, information on the choice of priors and Markov chain Monte Carlo settings
☒	☐	For hierarchical and complex designs, identification of the appropriate level for tests and full reporting of outcomes
☒	☐	Estimates of effect sizes (e.g. Cohen's *d*, Pearson's *r*), indicating how they were calculated

Our web collection on statistics for biologists contains articles on many of the points above.

Software and code

Policy information about availability of computer code

Data collection	EPU (Thermofischer Scientific)
Data analysis	RELION 4.0<br>CryoSparc v4.2.1<br>GraphPad Prism 8.0.1<br>CCP-EM v8.0<br>REFMAC5<br>PHENIX 1.17.1-3660<br>Coot 0.9.6.2<br>Chimera 1.16<br>Pymol 2.5.0<br>Molprobity (via PHENIX)<br>EMRinger (via PHENIX)<br>Gromacs 2020.3<br>ICM-Pro v3.9-2b molecular modeling and drug discovery suite<br>MDTraj software package

For manuscripts utilizing custom algorithms or software that are central to the research but not yet described in published literature, software must be made available to editors and reviewers. We strongly encourage code deposition in a community repository (e.g. GitHub). See the Nature Portfolio guidelines for submitting code & software for further information.

Data

Policy information about availability of data

All manuscripts must include a data availability statement. This statement should provide the following information, where applicable:

- Accession codes, unique identifiers, or web links for publicly available datasets
- A description of any restrictions on data availability
- For clinical datasets or third party data, please ensure that the statement adheres to our policy

All data within this manuscript is made available. The coordinates for the D3R:Gαβγ:scFv16 with the FOB02-04A in conformation A and B have been deposited in the Protein Data Bank with accession codes 9F33 [https://www.rcsb.org/structure/9f33] (Conformation A D3R:FOB02-04A structure) and 9F34 [https://www.rcsb.org/structure/9f34] (Conformation B D3R:FOB02-04A structure). The cryo-EM maps generated in this study have been deposited in the Electron Microscopy Data Bank (EMDB) with accession codes 50168 [https://www.emdataresource.org/EMD-50168] (Conformation A D3R:FOB02-04A cryo-EM map) and 50169 [https://www.emdataresource.org/EMD-50168] (Conformation B D3R:FOB02-04A cryo-EM map). The trajectories for the Molecular Dynamics simulations have been deposited as an open-access repo on Zenodo83: Nazarova, A. (2024). Molecular Dynamics Trajectories for the D3 receptor (D3R) complexes bound with a GαOβγ heterotrimer and 1) FOB02-04A bitopic agonist; 2) pramipexole. [Data set]. Zenodo. https://doi.org/10.21203/rs.3.rs-3433207/v1. A Source Data file is provided with this paper including (but not limited to) all functional data. Oligonucleotides used can be found in Supplementary Data 1.

Research involving human participants, their data, or biological material

Policy information about studies with human participants or human data. See also policy information about sex, gender (identity/presentation), and sexual orientation and race, ethnicity and racism.

Reporting on sex and gender	*Use the terms sex (biological attribute) and gender (shaped by social and cultural circumstances) carefully in order to avoid confusing both terms. Indicate if findings apply to only one sex or gender; describe whether sex and gender were considered in study design; whether sex and/or gender was determined based on self-reporting or assigned and methods used.<br>Provide in the source data disaggregated sex and gender data, where this information has been collected, and if consent has been obtained for sharing of individual-level data; provide overall numbers in this Reporting Summary. Please state if this information has not been collected.<br>Report sex- and gender-based analyses where performed, justify reasons for lack of sex- and gender-based analysis.*
Reporting on race, ethnicity, or other socially relevant groupings	*Please specify the socially constructed or socially relevant categorization variable(s) used in your manuscript and explain why they were used. Please note that such variables should not be used as proxies for other socially constructed/relevant variables (for example, race or ethnicity should not be used as a proxy for socioeconomic status).<br>Provide clear definitions of the relevant terms used, how they were provided (by the participants/respondents, the researchers, or third parties), and the method(s) used to classify people into the different categories (e.g. self-report, census or administrative data, social media data, etc.)<br>Please provide details about how you controlled for confounding variables in your analyses.*
Population characteristics	*Describe the covariate-relevant population characteristics of the human research participants (e.g. age, genotypic information, past and current diagnosis and treatment categories). If you filled out the behavioural & social sciences study design questions and have nothing to add here, write "See above."*
Recruitment	*Describe how participants were recruited. Outline any potential self-selection bias or other biases that may be present and how these are likely to impact results.*
Ethics oversight	*Identify the organization(s) that approved the study protocol.*

Note that full information on the approval of the study protocol must also be provided in the manuscript.

Field-specific reporting

Please select the one below that is the best fit for your research. If you are not sure, read the appropriate sections before making your selection.

☒ Life sciences ☐ Behavioural & social sciences ☐ Ecological, evolutionary & environmental sciences

For a reference copy of the document with all sections, see nature.com/documents/nr-reporting-summary-flat.pdf

Life sciences study design

All studies must disclose on these points even when the disclosure is negative.

Sample size	For cryo-EM studies, the size of the dataset was estimated based on the amount required to reach a relevant resolution in similar samples.<br>For signalling cell assays obtaining potency and efficacy, at three technical replicates with 3-5 independent biological replicates where performed. This design yields low errors and it is a standard in the field.<br>MD simulations were performed in replicates to ensure reproducibility.
Data exclusions	No exclusions were made.
Replication	Structure determination does not require replication because it represents the average structure of several hundred thousand molecules. All

Replication	biochemical assays had a number of independent experiments (= or >3) performed with technical triplicates . MD simulations were performed in replicates to ensure reproducibility.
Randomization	All variables could be controlled and so randomization was not required.
Blinding	No blinding was attempted or needed since biological replicates were sufficient.

Reporting for specific materials, systems and methods

We require information from authors about some types of materials, experimental systems and methods used in many studies. Here, indicate whether each material, system or method listed is relevant to your study. If you are not sure if a list item applies to your research, read the appropriate section before selecting a response.

Materials & experimental systems

n/a	Involved in the study
☐	☒ Antibodies
☐	☒ Eukaryotic cell lines
☒	☐ Palaeontology and archaeology
☒	☐ Animals and other organisms
☒	☐ Clinical data
☒	☐ Dual use research of concern
☒	☐ Plants

Methods

n/a	Involved in the study
☒	☐ ChIP-seq
☒	☐ Flow cytometry
☒	☐ MRI-based neuroimaging

Antibodies

Antibodies used	anti-Flag HRP conjugate (Sigma Aldrich, A8592)
Validation	The antibody was used to perform the quantification of surface expression of receptors variants on HEK293T cells. The antibody was validated using non-transfected HEK293 cells, and has been used in similar studies for equivalent purposes (e.g. 10.1016/j.molcel.2021.01.003)

Eukaryotic cell lines

Policy information about cell lines and Sex and Gender in Research

Cell line source(s)	HEK293T are commercial laboratory cell lines obtained from ATCC (CRL-3216 ™)<br>Trichoplusia ni are commercial cell lines purchased from Expressions Systems (94-002F)
Authentication	The cell lines were not authenticated by the authors as this was performed by the supplier.
Mycoplasma contamination	All cell lines were tested and confirmed as negative in mycoplasma contamination.
Commonly misidentified lines (See ICLAC register)	No commonly misidentified cell lines were used.

Plants

Seed stocks	*Report on the source of all seed stocks or other plant material used. If applicable, state the seed stock centre and catalogue number. If plant specimens were collected from the field, describe the collection location, date and sampling procedures.*
Novel plant genotypes	*Describe the methods by which all novel plant genotypes were produced. This includes those generated by transgenic approaches, gene editing, chemical/radiation-based mutagenesis and hybridization. For transgenic lines, describe the transformation method, the number of independent lines analyzed and the generation upon which experiments were performed. For gene-edited lines, describe the editor used, the endogenous sequence targeted for editing, the targeting guide RNA sequence (if applicable) and how the editor was applied.*
Authentication	*Describe any authentication procedures for each seed stock used or novel genotype generated. Describe any experiments used to assess the effect of a mutation and, where applicable, how potential secondary effects (e.g. second site T-DNA insertions, mosiacism, off-target gene editing) were examined.*
